# Xbp1 targets canonical UPR^ER^ and non-canonical pathways in separate tissues to promote longevity

**DOI:** 10.1016/j.isci.2024.109962

**Published:** 2024-05-11

**Authors:** Mengjia Li, Haocheng Shou, Guillermo Martínez Corrales, Tatiana Svermova, Alessandra Vieira Franco, Nazif Alic

**Affiliations:** 1 Institute of Healthy Ageing and the Research Department of Genetics, Evolution, and Environment, University College London, London WC1E 6BT, UK

**Keywords:** Biological sciences, Molecular biology, Molecular mechanism of gene regulation

## Abstract

Transcription factors can reprogram gene expression to promote longevity. Here, we investigate the role of *Drosophila* Xbp1. Xbp1 is activated by splicing of its primary transcript, *Xbp1*^*u*^, to generate Xbp1^s^, a key activator of the endoplasmic reticulum unfolded protein response (UPR^ER^). We show that Xbp1^s^ induces the conical UPR^ER^ in the gut, promoting longevity from the resident stem cells. In contrast, in the fat body, Xbp1^s^ does not appear to trigger UPR^ER^ but alters metabolic gene expression and is still able to extend lifespan. In the fat body, Xbp1^s^ and dFOXO impinge on the same target genes, including the PGC-1α orthologue Srl, and *dfoxo* requires *Xbp1* to extend lifespan. Interestingly, unspliceable version of the *Xbp1* mRNA, *Xbp1*^*u*^ can also extend lifespan, hinting at roles in longevity for the poorly characterized Xbp1^u^ transcription factor. These findings reveal the diverse functions of Xbp1 in longevity in the fruit fly.

## Highlights

• Xbp1^s^ triggers distinct transcriptional profiles in the gut and fat body
• Xbp1^s^ can extend lifespan independently from both tissues
• Fat body Xbp1 is essential for the dFOXO-induced lifespan extension
• Overexpressing the unspliceable Xbp1^U^ extends *Drosophila* lifespan

## Graphical abstract

**Figure:**
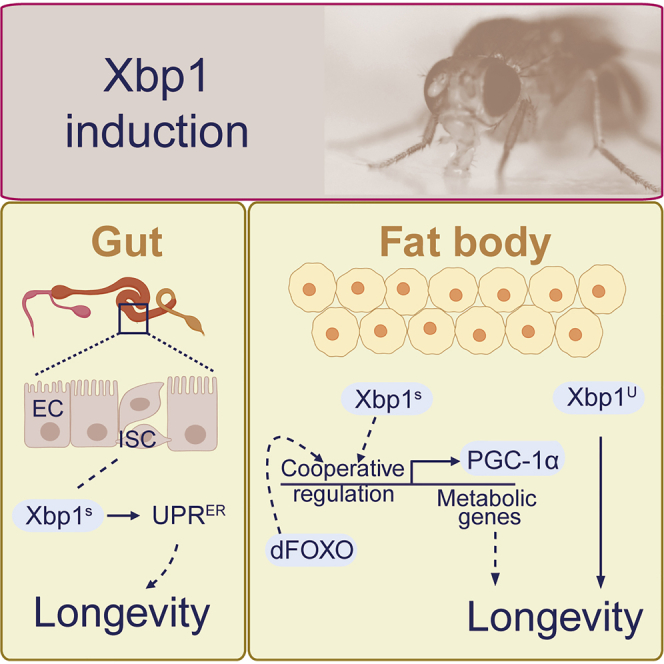

## Introduction

Aging can be observed as an inherent deterioration of a physiological system with time. The rate of aging is plastic and driven by tractable biological processes, such as loss of proteostasis and deregulated nutrient sensing, that are interconnected into a complex, interdependent network.^1^ Transcription factors (TFs) regulate gene expression and impact on a number of aging drivers, thus playing a crucial role in molding the longevity of an animal.^2^ For example, forkhead box O (FOXO) TFs have an evolutionary conserved role in regulating organismal stress responses and metabolism. Their activity can extend lifespan of many species including worms and flies, and they have been implicated in human aging.^3^^,^^4^^,^^5^^,^^6^Currently several pro-longevity TFs and TFs families, with evolutionarily conserved effects, are known across species^2^ but detailed mechanisms of how these TFs regulate the rate of aging have not been fully elucidated.

Xbp1 is an evolutionary conserved TF that acts in the IRE1 branch of the endoplasmic reticulum unfolded protein response pathway (UPR^ER^) and has a key role in maintaining cellular proteostasis.^7^^,^^8^^,^^9^ *Xbp1* mRNA has two splice variants. Upon ER stress, activated IRE1 catalyzes the unconventional splicing of *Xbp1*^*u*^ into *Xbp1*^*s*^, removing a 23 bp sequence to induce a frameshift allowing the translation of the highly active Xbp1^s^ TF.^9^ Subsequently, Xbp1^s^ activates the transcription of the genes involved in UPR^ER 7^.

Cellular ability to maintain the health of the proteome (proteostasis) declines with age, in part due to blunted activation and compromised capacity of the proteostasis-ensuring pathways, such as UPR^ER^.^10^^,^^11^ Indeed, studies in worms have shown that *Xbp1*^*s*^ overexpression solely in the intestine or pan-neuronally can increase lifespan.^10^ In both cases, Xbp1^s^ stimulates the activation of UPR^ER^ in older animals.^10^ The lifespan extension is coupled with a metabolic shift and increased lysosomal activity in the intestine, which acts in concert with UPR^ER^ activation to maintain proteostasis.^12^^,^^13^ Indeed, Xbp1 is also required for the longevity and improved ER stress resistance of a *daf2* mutant.^14^ Hence, Xbp1 with its canonical UPR^ER^ role contributes to longevity in worms.

Our recent work identified a pro-longevity effect of Xbp1 in *Drosophila*, where we found that *Xbp1*^*s*^ overexpression in the gut and fat body can extend lifespan.^15^ In the current study we further characterize the role of Xbp1 in fly longevity. Surprisingly, *Xbp1*^*s*^ induction triggered distinct gene expression programs in the two organs. Xbp1^s^’s activity in the gut aligned with its canonical role in activating UPR^ER^, and the activation of *Xbp1*^*s*^ solely in the intestinal stem cells was sufficient to increase lifespan. In the fat body, Xbp1^s^ regulated genes involved in metabolism and this activity was also sufficient to promote longevity. We show that dFOXO and Xbp1^s^ impinge on the same target genes in the fat body, including the Srl TF, with dFOXO requiring Xbp1 to extend lifespan. Lastly, we show that induction of an unspliceable form of Xbp1 can also extend lifespan.

## Results

### *Xbp1^s^* overexpression in the gut and fat body extends lifespan and healthspan

**Figure 1:**
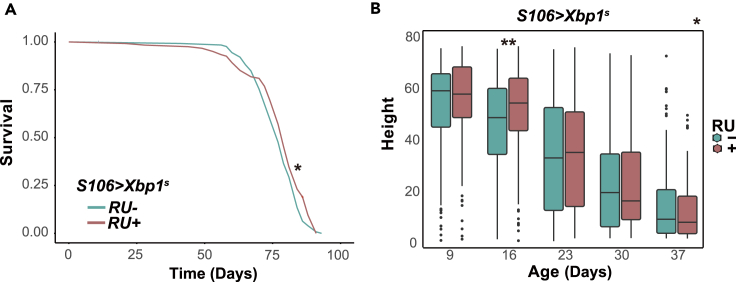
Overexpression of *Xbp1*^*s*^ in the gut and fat body promotes longevity and health (A) Lifespan assays of *S106>Xbp1*^*s*^ females fed or not RU^486^. RU^486^- (control) *n* = 126 dead/0 censored flies, RU^486^ + *n* = 121 dead/1 censored flies, median lifespan +2.6%, *p* = 0.04, *log rank test*. (B) Negative geotaxis assays of *S106>Xbp1*^*s*^. For RU^486^- (control), n varies from 120 flies (day 9) to 99 flies (day 37). For RU^486^+, n varies from 117 flies (day 9) to 99 flies (day 37). No significant effect of age-by- RU^486^ interaction, effect of age: *p* < 10^−16^, effect of RU^486^*p* = 0.026, *linear model (lm).* In pairwise comparisons, a significant effect of RU^486^ is detected on day 16 (*p* = 0.005) but no other days, one-tailed Student’s *t* test. Source data for all the main figures and related supplementary figures is available at Data S1. *∗p < 0.05, ∗∗p < 0.01*.

We initially confirmed previous observations^15^: the induction of *Xbp1*^*s*^ in the gut and fat body by RU^486^ feeding of *S106>Xbp1*^*s*^ adult females consistently promoted longevity in independent trials, even though the magnitude of the effect varied from 2.6% to 9.1%.^15^ (Figure 1A, also shown in Figure S1A), whereas RU^486^ feeding of the driver alone control had no such effect (Figure 1B). We extended this finding by examining whether Xbp1^s^ was also able to promote health in older flies. As in humans, fly’s neuromuscular performance declines with age. This decline can be observed as a decreased ability to climb a vertical surface in the negative geotaxis assay.^16^ We examined the climbing ability of *S106>Xbp1*^*s*^ females in the presence or absence of the inducer RU^486^ over a course of several weeks and analyzed the data using a *linear model* (*lm*). We found the *Xbp1*^*s*^ induction in the gut and fat body was sufficient to improve the flies’ climbing performance overall and mostly protected against climbing decline in the early stage of aging. (Figure 1B, observed as significant effect of RU^486^ in the *lm p* = 0.026; with significant difference in performance on day 16, *p* = 0.005, *t-test*). Thus, *Xbp1*^*s*^ overexpression not only promotes longevity but also improves aspects of fly health.

### *Xbp1*^*s*^ overexpression triggers ER stress response in the gut while affecting metabolic processes in the fat body

To elucidate the pro-longevity transcriptional program triggered by Xbp1^s^ activation, we profiled the gut and fat body transcriptomes of *Xbp1*^*s*^-overexpressing flies at day 9 using RNA-Seq. To remove any artifacts of RU^486^ feeding, we performed the same experiment on females carrying the driver alone (*S106* alone). Genes that responded to RU^486^ in this driver-alone control (1 in fat body and 24 in gut, all the differentially expressed gene lists are available in Data S1) were removed from subsequent analysis of the response to *Xbp1*^*s*^ induction.

**Figure 2:**
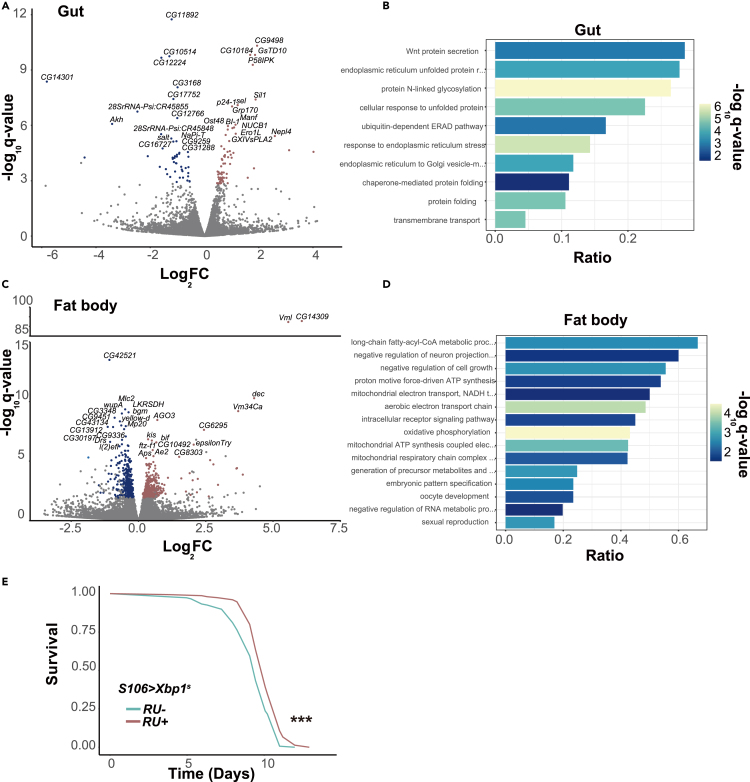
Induction of *Xbp1*^*s*^ elicits ER stress response in the gut while it impacts metabolic change in the fat body (A) Volcano plots showing the effect of *Xbp1*^*s*^ induction on transcripts in the gut with significantly upregulated genes (FDR 10%) shown in red and significantly downregulated genes shown in blue. The names of 15 most upregulated and 15 most downregulated genes (based on log_2_ fold change) are given. (B) Enriched biological process gene ontology (GO) terms (FDR 10%) for all the genes differentially expressed in the gut. (C) Volcano plots showing the effect of *Xbp1*^*s*^ induction on transcripts in the fat body, colored and annotated as in (A). (D) Enriched biological process GO terms (FDR 10%) for all the genes differentially expressed in the fat body. (E) Starvation assays of *S106>Xbp1*^*s*^ females fed or not RU^486^. RU^486^- (control) *n* = 148 dead/0 censored flies, RU^486^ + *n* = 150 dead/0 censored flies, *p* < 10^−5^, *log rank test*. Note in (A) – (D) the q value denotes the expected FDR. *∗∗∗p < 0.001*.

In the gut of *S106>Xbp1*^*s*^ females, we detected 114 genes that responded significantly to *Xbp1*^*s*^ overexpression (10% false discovery rate [FDR], Figure 2A). To understand the functions regulated by Xbp1^s^ in this organ, we performed Gene Ontology (GO) enrichment analysis. As expected, most of the genes regulated by Xbp1^s^ were involved in ER stress and the unfolded protein response (Figure 2B), in line with the canonical role of Xbp1.^7^

Surprisingly, we observed a distinct transcriptomic profile in the fat body, where we detected 772 differentially expressed genes of which only 3 were also differentially expressed in response to *Xbp1*^*s*^ in the gut (Figure 2C). Interestingly, the ER stress responsive genes that were differentially expressed in the gut did not appear transcriptionally activated in the fat body (Figure S2A). Rather, the set of 772 genes transcriptionally responsive to *Xbp1*^s^ in the fat body was enriched in pathways related to mitochondrial metabolism, DNA damage & cell cycle regulation (Figure 2D). With qPCR, we confirmed the upregulation in the fat body of three functionally important genes: the key mitochondrial metabolic regulator *srl,*^17^ the DNA-damage responsive Chk1 (*grp*) and Chk2 (*lok*) kinases^18^ (Figures S2B and S2C; see later section for *srl*). We also examined if there are any phenotypes of *Xbp1*^*s*^ induction that are consistent with an impact on metabolism. Indeed, we found that *Xbp1*^*s*^ induction resulted in starvation resistance (Figure 2E). Overall, the transcriptional programs triggered by *Xbp1*^*s*^ overexpression appear to impact different functions in different organs: a classic ER stress response was observed in the gut while changes relating to metabolism and other non-conical functions were dominant in the fat body.

### Xbp1^s^ promotes longevity independently from both the intestinal stem cells and the fat body

**Figure 3:**
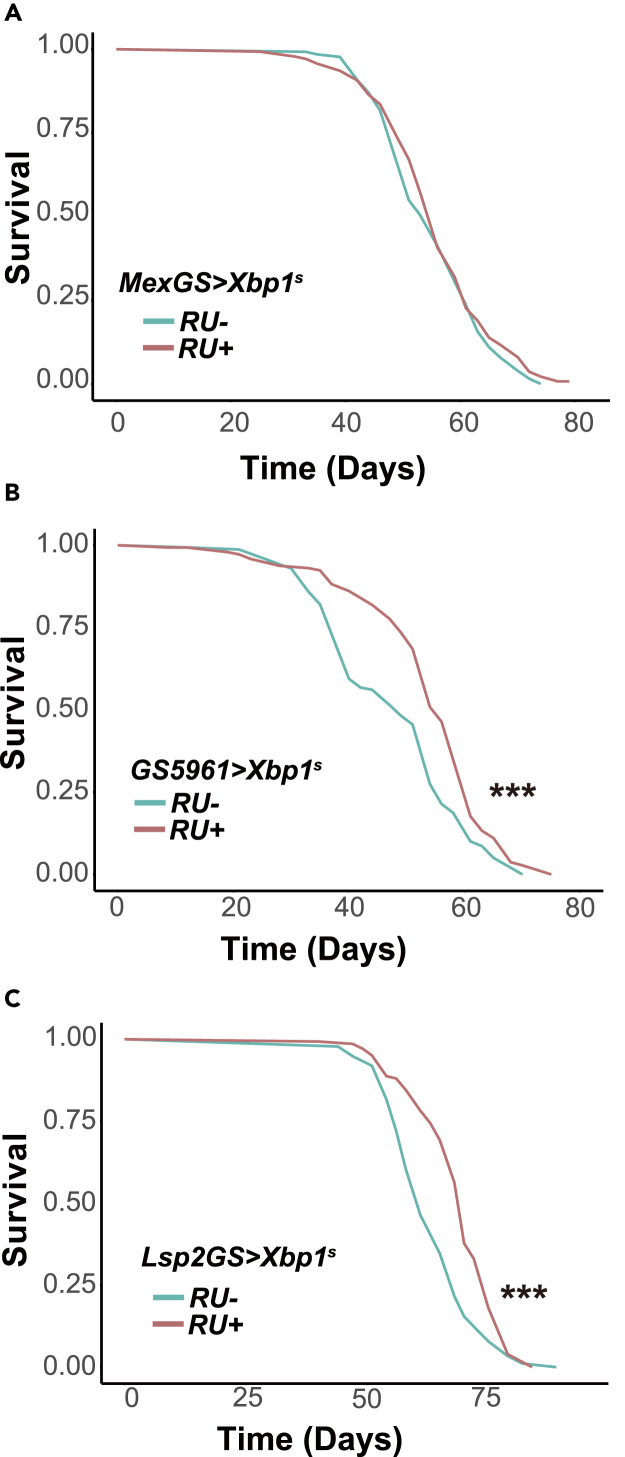
*Xbp1*^*s*^ induction in either the intestinal stem cell or the fat body is sufficient to extend lifespan (A) Lifespans assays of *MexGS>Xbp1*^*s*^ females fed or not RU^486^. RU^486^- (control) *n* = 130 dead/1 censored flies, RU^486^ + *n* = 138 dead/2 censored flies, *p* = 0.246, *log rank test*. (B) Lifespans assays of *GS5961>Xbp1*^*s*^ females. RU^486^- (control) *n* = 152 dead/4 censored flies, RU^486^ + *n* = 138 dead/7 censored flies, median lifespan +14.3%, *p* = 1.85 × 10^−5^, *log rank test*. (C) Lifespans assays of *Lsp2GS > Xbp1*^*s*^ females. RU^486^- (control) *n* = 122 dead/14 censored flies, RU^486^ + *n* = 132 dead/11 censored flies, median lifespan +14.8%, *p* = 3.2 × 10^−7^, *log rank test*. *∗∗∗p < 0.001*.

Proteostasis has been associated with animal longevity.^11^ As Xbp1^s^ appeared to regulate the ER stress response in the gut, we tested if induction of *Xbp1*^*s*^ solely in the gut was sufficient to extend lifespan. The most abundant cells in the gut are the enterocytes (EC) that perform absorption. However, overexpressing *Xbp1*^*s*^ with the enterocyte-specific, inducible driver MexGS^19^ did not extend lifespan (Figure 3A, driver-alone control in Figure S3A). On the other hand, recent studies have shown that the loss of protein homeostasis in the intestinal stem cell (ISC) limits the longevity of female flies.^20^ We tested the effect of overexpressing *Xbp1*^*s*^ with the GS5961 driver, which has been characterized as driving gene expression solely from the ISC.^21^ Indeed, we found that *Xbp1*^*s*^ overexpression in the ISC was sufficient to extend lifespan (Figure 3B, driver-alone control in Figure S3B).

We also tested the pro-longevity effect of *Xbp1*^*s*^ overexpression with Lsp2GS,^22^ a driver whose expression specifically in the adult fat body is produced by the well-characterized promoter of the *Lsp2* gene.^23^ Given that the fat body transcriptome profile was different from the gut, and with no indication of activated ER proteostasis response, it was quite interesting that the fat body-specific *Xbp1*^*s*^ overexpression could also extend lifespan (Figure 3C, driver-alone control in Figure S3C). Hence, even though *Xbp1*^*s*^ induction activates distinct transcriptional programs in the two organs, it is able to extend lifespan from both.

### *Xbp1*^*s*^ and *dfoxo* drive similar transcriptional programs in the fat body

**Figure 4:**
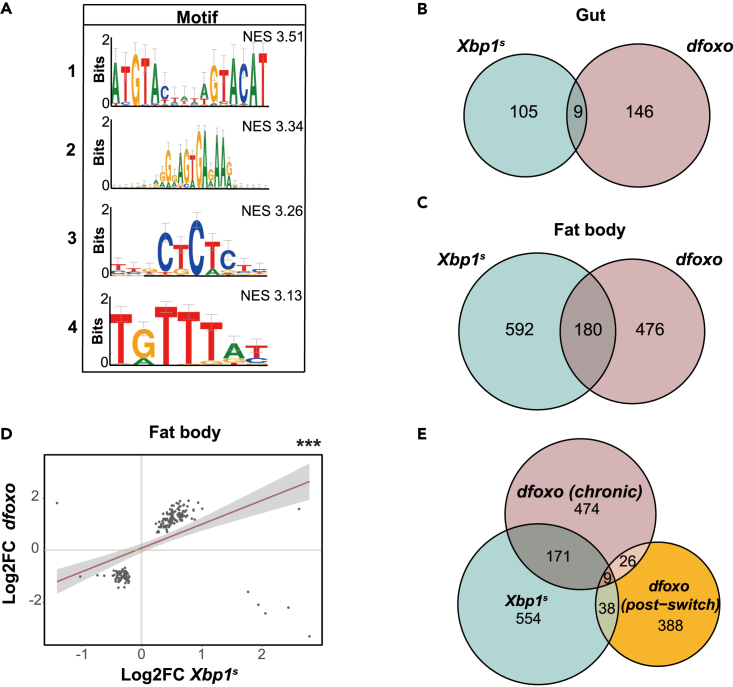
*Xbp1*^*s*^ induction and *dfoxo* induction trigger similar transcriptional response in the fat body (A) Motifs identified as enriched within the promoters of genes differentially expressed after *Xbp1*^*s*^ induction in the fat body. Motif 1 sequence is similar to that typically bound by BtbVII, Motif 2 was related to Sirt6 binding motif, Motif 3 was related to that of Trl, and Motif 4 was related to that bound by dFOXO. NES – Normalized Enrichment Score. (B) Overlap of genes differentially expressed upon *Xbp1*^*s*^ induction and *dfoxo* induction in the guts. *p* < 10^−6^, *one-sided hypergeometric test*. (C) Overlap of genes differentially expressed upon *Xbp1*^*s*^ induction and *dfoxo* induction in the fat body. *p* < 10^−90^, *one-sided hypergeometric test*. (D) Relationship between log_2_FC in gene expression caused by *Xbp1*^*s*^ induction and that caused *dfoxo* induction in the fat body. There is strong correlation for genes differentially expressed in both conditions (180 overlapping genes from B) (*linear regression*, *β =* 0.22, *p* = 1.79 ∗ 10^−10^). (E) Overlap of genes differentially expressed upon *Xbp1*^*s*^ induction, *dfoxo* induction (chronic), *dfoxo* post-switch in the fat body. *∗∗∗p < 0.001*.

The non-canonical role of *Xbp1*^*s*^ in the fat body and its ability to promote longevity prompted us to further explore its function in this organ. Firstly, we performed motif enrichment analysis on promoters of genes differentially regulated by *Xbp1*^*s*^ in the fat body to find potential Xbp1^s^ partners. We found that the dFOXO binding motif was enriched (Figure 4A). This was interesting as *dfoxo* also promotes longevity when overexpressed in the gut and fat body; it can do this when induced chronically, throughout adulthood,^4^^,^^24^ as well as when its induction is confined to the first three weeks of adulthood and then switched off (termed *dfoxo-switch*).^15^ Transient induction of *dfoxo* (*dfoxo-switch*) results in subsequent induction of *Xbp1*^*s*^ and indeed sole expression of *Xbp1*^*s*^ in later adulthood is sufficient to extend lifespan.^15^ On the other hand, the relationship between Xbp1 and dFOXO during chronic expression of *dfoxo* has not been thoroughly examined.

To elucidate the potential links between *dfoxo* and *Xbp1* during chronic induction, we took advantage of our previously published datasets.^25^ We used the RNA-Seq data that were obtained from flies in which *dfoxo* was induced in the same experimental setup that we used here (day 9 females after 7 days of *dfoxo* induction). We compared the transcriptome profiles of *dfoxo* overexpression and *Xbp1*^*s*^ overexpression in the gut and fat body separately. For the gut, only 9 genes were significantly differentially expressed in both conditions (Figure 4B). The overlap itself was significant (*p* < 10^−6^) but these genes did not change overall in the same direction in response to *Xbp1*^*s*^ and *dfoxo* (Figure S4A), indicating the two TFs do not act in concert in this organ.

On the other hand, there were 180 genes differentially regulated by both *dfoxo* and *Xbp1*^*s*^ overexpression in the fat body (Figure 4C) and this overlap was highly significant (*p* < 10^−90^). Notably, 96.7% (174 of 180) of these overlapping genes changed in the same direction (Figure 4D), indicating a shared transcriptional outcome for these two TFs in the fat body. We further compared the transcriptional profile characterized from the *Xbp1* null mutant larvae^26^ with that observed upon *dfoxo* overexpression in the fat body. A strong negative correlation was observed for the genes differentially expressed in both conditions (Figure S4B), indicating *Xbp1* and *dfoxo* act on a shared set of genes to promote their expression in the same direction, i.e. drive a similar expression program, during chronic induction.

Is this common transcriptional program any different from that observed after *dfoxo-switch*^15^? We compared the transcriptional profiles observed during chronic induction of *Xbp1*^*s*^, *dfoxo* and after *dfoxo-switch* to explore if the 180 Xbp1^s^/dFOXO shared targets were also altered by the *dfoxo-switch* (Figure 4E). We found only 9 genes in the overlap. Furthermore, separating these by their direction of change, we found none of the shared targets responded to *dfoxo-switch* (Figures S4C and S4D) as they do to chronic expression of both *Xbp1*^*s*^ and *dfoxo,* suggesting that the transcriptional program observed after *dfoxo-switch* did not engage Xbp1^s^/dFOXO shared targets in the same way that chronic expression of either TF does. Hence, the relationship between dFOXO and Xbp1^s^ during chronic induction is likely distinct to that after *dfoxo-switch*.

### The pro-longevity effect of *dfoxo* requires *Xbp1*

**Figure 5:**
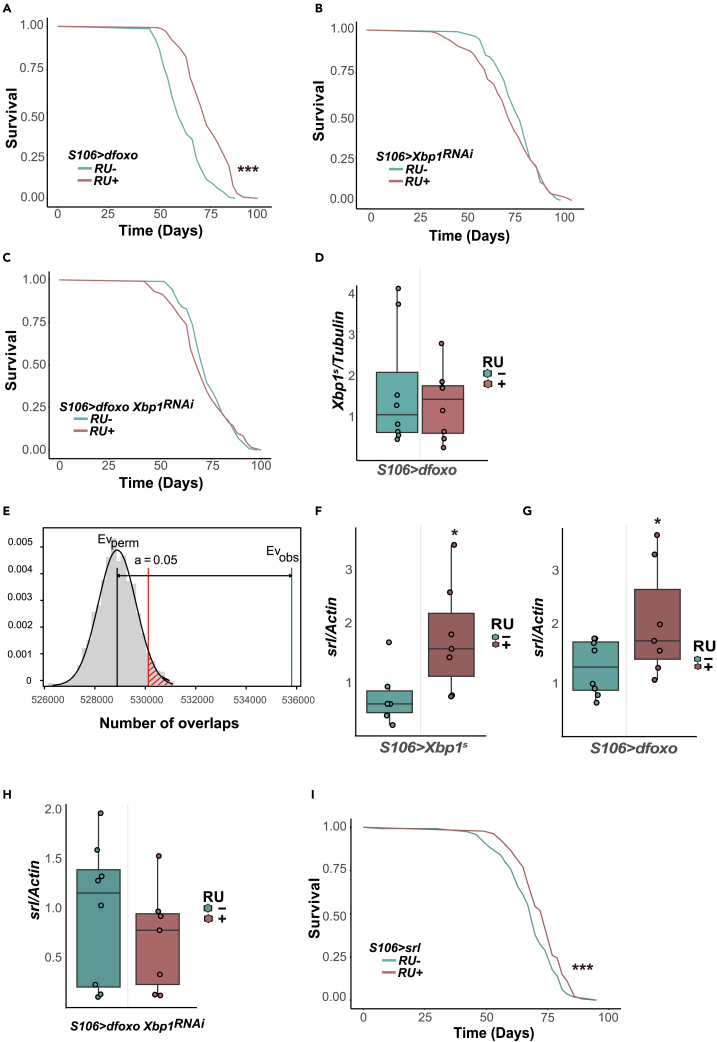
*dfoxo* requires *Xbp1* to extend lifespan (A) Lifespan assays of *S106>dfoxo* females fed or not RU^486^. RU^486^- (control) *n* = 144 dead/2 censored flies, RU^486^ + *n* = 140 dead/0 censored flies, median lifespan +23.7%, *p* < 2 x 10^−16^, *log rank test*. (B) Lifespan assays of *S106>Xbp1*^*RNAi*^ females. RU^486^- (control) *n* = 107 dead/1 censored flies, RU^486^ + *n* = 105 dead/2 censored flies, *p* = 0.3, *log rank test*. (C) Lifespan assays of *S106>dfoxo Xbp*^*1RNAi*^ females. RU^486^- (control) *n* = 106 dead/0 censored flies, RU^486^ + *n* = 118 dead/1 censored flies, *p* = 0.4, *log rank test*. (D) qPCR quantification of *Xbp1*^*s*^ transcript upon *dfoxo* induction in the fat body (8 ≥ *n* ≥ 7). *p* = 0.20, one-tailed Student’s *t* test. (E) Bootstrap analysis of the co-occurence of Xbp1^s^-bound motif (ACGT) and dFOXO-bound motif (TGTTT) in the fly genome. The Ev_obs_ is the exact number of co-occurrences of the two motifs within 200 bp. The Ev_perm_ represents the mean of distribution of the bootstrapping, indicating the probability of co-occurrence of random sequences of the same sizes within the regions. (F) qPCR quantifications of *srl* transcripts in *Xbp1*^*s*^-induced (*n* = 6, *p* = 0.042) fat bodies at day 9. One-tailed Student’s *t* test. (G) qPCR quantifications of *srl* transcripts in *dfoxo*-induced (8 ≥ *n* ≥ 7, *p* = 0.019) fat bodies at day 9. One-tailed Student’s *t* test. (H) qPCR quantification of *srl* transcript upon *dfoxo* induction in epistasis with *Xbp1*^*RNAi*^ in the fat body (8 ≥ *n* ≥ 7). *p* = 0.20, one-tailed Student’s *t* test. (I) Lifespan assays of *S106>srl* females fed or not RU^486^. RU^486^- (control) *n* = 115 dead/18 censored flies, RU^486^ + *n* = 135 dead/1 censored flies, median lifespan +5.7%, *p* = 0.0041, *log rank test*. *∗p < 0.05, ∗∗∗p < 0.001*.

Given the links between dFOXO and Xbp1 we wanted to examine the functional interplay of these two pro-longevity TFs in the context of aging. As expected, chronic *dfoxo* overexpression in the gut and fat body with the *S106* driver promoted longevity (Figure 5A). We knocked *Xbp1* down using a validated RNA-mediated interference (RNAi) construct,^27^ which itself did not alter lifespan (Figure 5B). Interestingly, *Xbp1* downregulation abolished the pro-longevity effect of *dfoxo* (Figure 5C) and we confirmed with Cox Proportional Hazards analysis that the response to *dfoxo* induction by RU^486^ was significantly altered by the presence of *Xbp1*^*RNAi*^ (RU486 by genotype interaction, *p* = 2.72 x 10^−9^). Hence, *Xbp1* is required by *dfoxo* to promote longevity.

One simple explanation of these results would be that *dfoxo* induction upregulates the expression or activation of Xbp1. We tested this possibility by measuring the transcripts levels of both *Xbp1*^*s*^ and *Xbp1*^*u*^ during chronic *dfoxo* induction. Here, we found no change in expression levels and no change in the proportion of spliced *Xbp1* (Figures 5D and S5A). Corroborating this, we noticed that dFOXO binding motif is not found in the *Xbp1* promoter (−400 to +100 TSS); nor has dFOXO binding been detected in proximity of the *Xbp1* gene in a previous study.^28^ Again, this is distinct from the observed Xbp1 induction after *dfoxo-switch.*^15^ Note that, conversely, we also did not find any evidence that Xbp1 can regulate dFOXO on either mRNA or protein levels (Figures S5B–S5D).

We hypothesized that these two TFs tend to bind the same genome regions to regulate transcription cooperatively. We computationally tested this idea by estimating the co-occurrence of the Xbp1 binding motif and dFOXO binding motif in the genome. Indeed, we found their co-occurrence is higher than expected by chance (Figure 5E). Overall, our findings are consistent with a cooperation rather than cross-regulation existing between the two TFs and this cooperation appears essential for the longevity effects of dFOXO.

Within the 180 shared targets, *srl*, a key mitochondrial metabolic regulator,^17^ triggered our interest for further investigation as it has been associated with longevity in *Drosophila.*^29^^,^^30^ FOXO is known to bind to the PGC-1α (Srl orthologue in mammals) promoter and activate its expression in mammalian cells^31^; there is a clear dFOXO binding motif in the promoter of *Drosophila srl*. We confirmed that *srl* was upregulated by either *Xbp1*^*s*^ or *dfoxo* induction (Figures 5F and 5G). Furthermore, *Xbp1* knockdown abolished the upregulation of *srl* upon *dfoxo* overexpression (Figure 5H). *srl* overexpression with the S106 driver was sufficient to extend lifespan (Figure 5I). Overall, our data indicate that dFOXO extends lifespan through a cooperation with Xbp1, with their regulation of a common target, *srl,* likely explaining at least some of their pro-longevity effects.

### Unspliceable-*Xbp1* (*Xbp1*^*U*^) overexpression also promotes longevity from the fat body

**Figure 6:**
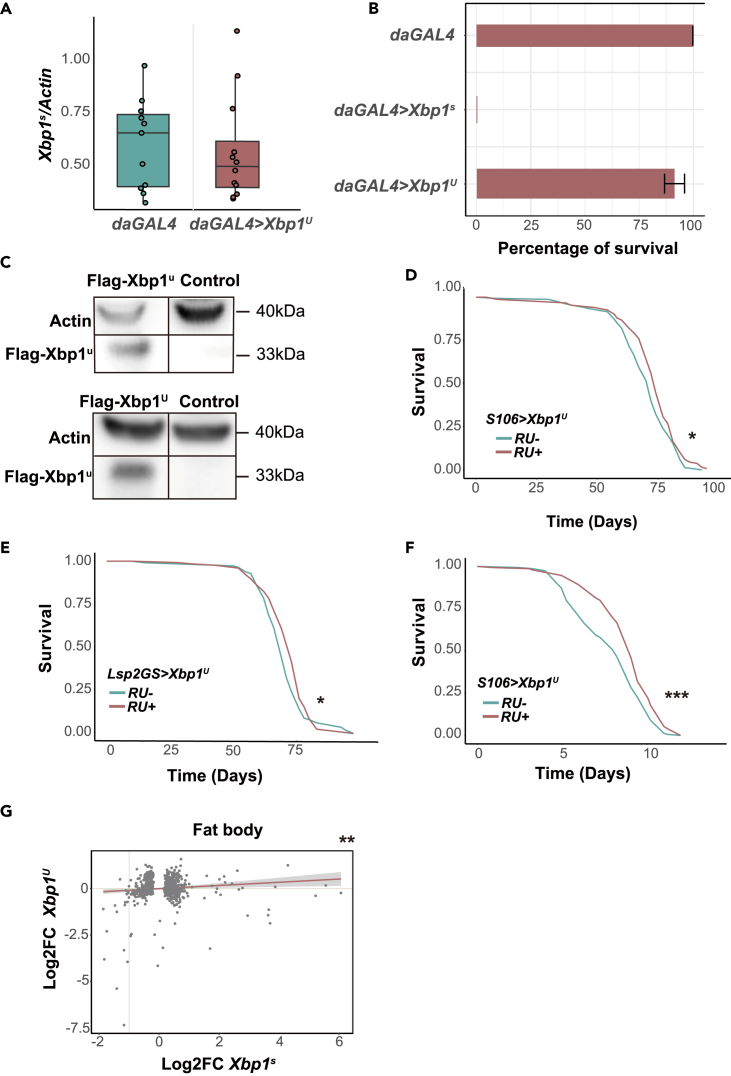
*Xbp1*^*U*^ induction in the fat body extends *Drosophila* lifespan (A) qPCR quantification of *Xbp1*^*s*^ transcript in *Xbp1*^*U*^-induced flies (*daGAL4>Xbp1*^*U*^) or *daGAL4* controls (12 ≥ *n* ≥ 11). *p* = 0.64, one-tailed Student’s *t* test. (B) Developmental lethality assays showing the percentage of adults merged from eggs. Data are represented as mean ± SEM based on the binomial distribution. *daGAL4 n* = 23, *daGAL4 > Xbp1*^*s*^*n* = 28, *daGAL4 > Xbp1*^*U*^*n* = 37. (C) Representative western blots against Flag-Xbp1^u^, in S2 cells 7 days after transfection of Act5c-Flag-Xbp1^U^ plasmid or control transfections. Images of membrane are shown in Figures S5F and S5G. (D) Lifespan assays of *S106>Xbp1*^*U*^ females fed or not with RU^486^. RU^486^- (control) *n* = 155 dead/0 censored flies, RU^486^ + *n* = 149 dead/3 censored flies, median lifespan +2.8%, *p* = 0.018, *log rank test*. (E) Lifespan assays of *Lsp2GS > Xbp*1^U^ females. RU^486^- (control) *n* = 110 dead/13 censored flies, RU^486^ + *n* = 122 dead/5 censored flies, median lifespan +5.8%, *p* = 0.047, *log rank test*. (F) Starvation assays of *S106>Xbp1*^*U*^ females. RU^486^- (control) *n* = 155 dead/0 censored flies, RU^486^ + *n* = 150 dead/0 censored flies, *p* = 0.0001, *log rank test*. (G) Relationship between log_2_FC in gene expression caused by *Xbp1*^*s*^ induction and *Xbp1*^*U*^ induction in the fat body. There is strong correlation for genes differentially expressed in either condition (*linear regression*, *β =* 0.11, *p* = 0.006). Note, however, that only a few genes are significantly affected by the induction of *Xbp1*^*U*^. *∗p < 0.05, ∗∗p < 0.01, ∗∗∗p < 0.001*.

During the course of this work we also examined the potential role of Xbp1^u^ in longevity. Xbp1^u^ protein arises from the primary, unspliced *Xbp1*^*u*^ transcript. It shares the DNA binding domain with Xbp1^s^ and recent studies have shown that Xbp1^u^ does have physiological roles on its own.^32^^,^^33^ A fly Xbp1^U^ construct has been generated that cannot be spliced into Xbp1^s^, due to a 2 bp mutation prior to the splicing site, which prevents IRE1-dependent splicing.^34^ We confirmed the 2 bp mutation by DNA sequencing (data not shown) and confirmed that ubiquitous *Xbp1*^*U*^ overexpression during development did not cause lethality, unlike the overexpression of *Xbp1*^*s*^, and that it did not produce a detectable increase in *Xbp1*^*s*^ levels (Figures 6A and 6B). The induction of the *Xbp1*^*U*^ increased the *Xbp1*^*u*^ mRNA levels *in vivo* (Figure S6A) and produced a protein of the expected size in S2 cells (Figures 6C, S6G, and S6H). To test the physiological role of Xbp1^u^ in the aging process, we overexpressed the above-described construct in the gut and the fat body and found that it can extend lifespan (Figure 6D, driver-alone control in Figure S6B). Additionally, we found that *Xbp1*^*U*^ overexpression only showed the beneficial effect when induced in the fat body; when induced in the ISCs lifespan was shortened (Figures 6E, S6C, and S6D). Consistent with a role in the fat body, Xbp1^U^ could improve starvation resistance as well as Xbp1^s^ did (Figure 6F).

We hypothesize that Xbp1^u^ may also function as a TF, so we characterized the transcriptome profiles in the gut and fat body of *Xbp1*^*U*^-overexpressing females. However, in both tissues we detected very limited gene expression changes (Figures S6E and S6F, differential expressed gene list in Data S1). This may result from weak transactivation activity that has been observed for Xbp1^u^.^9^ Even though the transcriptional changes in the fat body were correlated for the *Xbp1*^*s*^ and *Xbp1*^*U*^ overexpression (Figure 6G), the small number of significantly altered genes does not provide substantial evidence that *Xbp1*^*u*^ influences lifespan solely through its transcriptional activity. Still, our findings reveal an unexpected role for Xbp1^u^ in longevity.

## Discussion

In this study we show that *Xbp1*^*s*^ overexpression can induce tissue-specific transcriptional responses, with proteostasis-related genes regulated in the gut while genes encoding metabolic and other function were predominately regulated in the fat body. Apart from the classical UPR^ER^ regulation, the role of Xbp1 in metabolism and other aspects of cell function are increasingly described in different species and cell types.^13^^,^^35^^,^^36^^,^^37^^,^^38^ Our results indicate that the two sets of functions of Xbp1^s^ also occur in the fruit fly where they are separable by tissue.

Importantly, our findings highlight that distinct functions of Xbp1^s^ in separate tissues can independently promote longevity. Note that the increase in lifespan observed can vary between trials (e.g., see Figure S1A) due to an inherent variability of lifespan assays; for this reason we are reluctant to conclude that Xbp1 activity in either gut or fat body is more important for longevity. In previous studies, proteostasis maintenance in the intestine has been largely associated with the pro-longevity effects of Xbp1^s^.^10^^,^^20^ Our study is in line with these findings where proteostasis-promoting transcriptional response is linked to longevity specifically in the intestinal stem cells. On the other hand, little was known if Xbp1^s^-induced metabolic genes alone can modulate lifespan. Interestingly, our results indicate that in the fruit fly fat body, Xbp1^s^ induction is likely to promote longevity through non-canonical physiological functions of Xbp1^s^, such as metabolic regulation, and without an explicit activation of the classical UPR^ER^ response.

The fruit fly fat body is functionally equivalent to the mammalian adipose tissue and liver.^23^ In mice, adipose-specific overexpression of *Xbp1*^*s*^ can improve glucose and lipid metabolism and increase insulin insensitivity,^39^^,^^40^ indicating an evolutionary conservation of the beneficial, metabolic effects of Xbp1^s^ activity in the adipose tissues. However, the link to mammalian lifespan remains unclear: one study measured the lifespan of male mice with or without adipose-specific *Xbp1*^*s*^ induction, and no difference was observed^40^; however, the effect of Xbp1^s^ might be female-specific or triggered from other organs.

Furthermore, we highlight a Xbp1^s^-dFOXO interplay in the fat body where life-long, chronic induction of *dfoxo* requires *Xbp1* for lifespan extension. Importantly, the two pro-longevity TFs appear to share a large number of target genes and have highly similar transcriptional outcomes in the fat body. This adds an additional layer to previous findings where Xbp1 was observed to negatively regulate FOXO^41^^,^^42^^,^^43^; or our previous work that identified a TF relay where transient activation of *dfoxo* results in subsequent *Xbp1* induction.^15^ Note that Xbp1 induction was not observed during chronic activation of dFOXO. The findings we describe here are consistent with observations in worms where Xbp1 was required for lifespan extension in the context of a *daf2* loss of function,^14^ in which Xbp1 was similarly found to cooperate with DAF-16 (the worm FOXO orthologue) to induce a pro-longevity gene expression program. The worm study identified one gene that was transcriptionally co-regulated by the two TFs, and computationally suggested another 17 shared targets. Our study further revealed that the two TFs have a large proportion of shared targets but only in a specific tissue. We also present evidence that their interaction is not based on transcriptional cross-regulation but cooperation on shared targets across the genome.

Interestingly, previous studies have found a number of other TFs engaged in an interplay with dFOXO in the *Drosophila* fat body. E Twenty Six (ETS) TFs, Aop and Pnt, are antagonistic to each other and interact with dFOXO’s transcriptional output to determine lifespan.^25^^,^^28^ Indeed, *Xbp1*^*s*^ induction causes a transcriptome change similar to that caused by Aop activation and Pnt inhibition in the fat body (Data S1). Our current work, as well as that of others,^29^^,^^30^^,^^44^ adds Srl as another TF whose activity in the fat body promotes longevity, and which is transcriptionally activated by Xbp1^s^ and dFOXO. These 5 TFs are likely engaged in a transcriptional network in the *Drosophila* fat body, one that can impact whole animal longevity and whose further study may contribute to our understanding of regulatory networks that underly the plasticity of animal physiology.

Lastly, we found that Xbp1^u^, the less studied isoform of Xbp1, was also able to promote longevity in *Drosophila*. Xbp1^u^ has been linked to regulation of Xbp1^s^ and FOXO4 levels,^45^^,^^46^ metabolism and cell cycle regulation^33^^,^^34^. Most of these Xbp1^u^ functions are conducted through protein-protein interaction and independent of Xbp1^s^. Similarly, we found that Xbp1^u^, unlike Xbp1^s^, only regulated lifespan in the fat body. This implies different mechanism of Xbp1^u^-dependent lifespan regulation. Indeed, we characterized the Xbp1^u^ transcriptome, which showed few significant transcriptional changes upon induction in both tissues. The finding was in line with the previously observation of Xbp1^u^, which can bind to the DNA but only activate gene expression weakly.^7^ Further investigation of the function of Xbp1^u^ in cell and animal physiology may be warranted.

### Limitations of the study

Due to the lack of an anti-*Drosophila* Xbp1 antibody, we could not attempt to characterize genome-wide binding of Xbp1^s^ to examine if dFOXO and Xbp1^s^ share binding locations. Instead, we applied computational methods to suggest their cooperation was based on co-binding of targets across the genome. Additional experiments are also required to understand the pro-longevity effect of Xbp1^u^.

## STAR★Methods

### Key resources table

**Table:** 

REAGENT or RESOURCE	SOURCE	IDENTIFIER
**Antibodies**		

Rabbit anti-dFOXO	Giannakou et al.^4^	N/A
Mouse anti-FLAG	Abcam	Cat#F3165; RRID: AB_259529
Mouse anti-Actin	Abcam	Cat#ab8224; RRID: AB_449644
Goat anti-Rabbit IgG H&L (HRP)	Abcam	Cat#ab6721; RRID: AB_955447
Goat anti-Mouse IgG H&L (HRP)	Abcam	Cat#ab6789; RRID: AB_955439

**Chemicals, peptides, and recombinant proteins**		

TRIzol™ Reagent	Thermo Fisher Scientific	Cat#15596026
Schneider’s Drosophila medium	Sigma	Cat#S0146
NuPAGE™ 10%, Bis-Tris gel	Thermo Fisher Scientific	Cat#NP0316BOX
Nitrocellulose blotting membrane	GE Healthcare Life Sciences	Cat#10600003
Crescendo	Merck	Cat##WBLUR0500
SYBR™ Green PCR Master Mix	Applied Biosystems	Cat#4385612
Phusion™ High–Fidelity DNA Polymerase	Thermo Fisher Scientific	Cat#F530L
pENTR™/D-TOPO® gateway	Thermo Fisher Scientific	Cat#450218
Gateway™ LR Clonase™ II Enzyme Mix	Thermo Fisher Scientific	Cat#11791-020
Fetal Bovine Serum	Thermo Fisher Scientific	Cat#A3160801
Effectne Transfection reagent	Qiagen	Cat##301425
SuperScript™ II Reverse Transcriptase	Thermo Fisher Scientifc	Cat#18064014

**Deposited data**		

RNA-sequencing data	Gene Expression Omnibus	GSE246232

**Experimental models: Cell lines**		

Drosophila Schneider 2 (S2) cells	This lab	N/A

**Experimental models: Organisms/strains**		

D. melanogaster: wDah	This lab/Linda Partridge	N/A
D. melanogaster: S106 driver	This lab/Linda Partridge	N/A
D. melanogaster: GS5691 driver	Biteau et al.^21^	N/A
D. melanogaster: MexGS driver	Soule et al.^19^	N/A
D. melanogaster: Lsp2GS driver	Ragheb et al.^22^	N/A
D. melanogaster: UAS-dfoxo	Giannakou et al.^4^	N/A
D. melanogaster: UAS-dxbp1S	Huang et al.^26^	Gift from Prof. Hyung Don Ryoo
D. melanogaster: UAS-dxbp1U	Huang et al.^26^	Gift from Prof. Hyung Don Ryoo
D. melanogaster: UAS-dxbp1RA	Huang et al.^26^	Gift from Prof. Hyung Don Ryoo
D. melanogaster: daughterlessGAL4 driver	Bloomington Drosophila Stock Center	RRID:BDSC_55850
D. melanogaster: UAS-dxbp1 RNAi	Bloomington Drosophila Stock Center	RRID:BDSC_25990

**Oligonucleotides**		

Xbp1s F 5′ CCGAACTGAAGCAGCAACAGC 3	Martínez Corrales et al.^15^	N/A
Xbp1s_R 5′ GTATACCCTGCGGCAGATCC 3′	Martínez Corrales et al.^15^	N/A
Xbp1u F 5′ CAGCATCCAAAGCTGACCC 3′	Martínez Corrales et al.^15^	N/A
Xbp1u R 5′ GTAGGCAGAGGGCCACAAC 3′	Martínez Corrales et al.^15^	N/A
dFOXO F 5′ CCACCATGCTCGCTGGTCTC 3′	Alic et al.^28^	N/A
dFOXO R 5′ CTCCATTTGACTGACTGACTTG 3′	Alic et al.^28^	N/A
Srl F 5′ GGATTCACGAATGCTAAATGTGTTCC 3′	N/A	N/A
Srl R 5′ GATGGGTAGGATGCCGCTCAG3′	N/A	N/A
Actin F 5′ CACACCAAATCTTACAAAATGTGTGA 3′	Martínez Corrales et al.^15^	N/A
Actin R 5′ AATCCGGCCTTGCACATG 3′	Martínez Corrales et al.^15^	N/A
Full list of primers are available in the Source data		

**Software and algorithms**		

R and R studio (version R-4.2.2)	The R Project for Statistical Computing	https://www.r-project.org/
Excel (version 2403)	Microsoft	https://www.microsoft.com/en-gb/microsoft-365/excel
ImageJ (version 1.53t)	ImageJ	https://imagej.net/ij/download.html

**Other**		

DrosoFlippers	Drosoflipper	www.drosoflipper.com
QuantStudio 6cFlex real-time PCR machine	Thermo Fisher Scientific	N/A
Nanodrop 2000C spectrophotometer	Thermo Fisher Scientific	N/A
Glass beads, acid washed	Sigma	Cat#G8772
Skim Milk Powder	Millipore	Cat#70166

### Resource availability

#### Lead contact

Further information and requests for resources and reagents should be directed to and will be fulfilled by the lead contact, Nazif Alic (n.alic@ucl.ac.uk).

#### Materials availability

This study did not generate new unique reagents.

#### Data and code availability

• Data: All the reported RNA-seq data have been deposited at GEO. Accession numbers are listed in the key resources table. Source data of experiments reported in the paper is available in the supplemental figures and Data S1.
• Code: This paper does not report the original code.
• Any additional information required to reanalyze the data reported in this paper is available from the lead contact upon request.

### Experimental model and study participant details

#### Animals

All experiment were conducted with the wild-type Dahomey background stock, which was originally collected in 1970 in Dahomey and has since been maintained in population cages. All transgenes used in the study were backcrossed for at least 6 generation to this outbred, Dahomy background which carried a *w*^*1118*^ mutation and was Wolbachia-free (wDah). Flies were maintained in 25°C, 60% humidity with 12 h:12 h dark and light cycle. Standard sugar/yeast/agar (SYA) food was used for stock maintenance. Experiments were performed with female flies.

#### Cell lines

Drosophila S2 cells were were cultured in Schneider’s medium (Gibco/Thermo Scientific #21720024) supplied with 10% FBS (Gibco/Thermo Scientific #A3160801). Cells were maintained in 25°C incubator with 0% CO_2_ concentration and split into fresh media every week.

### Method details

#### Experiment setup, lifespan assays and starvation assays

For all physiological experiments, crosses were set up with males and females of the required genotypes in cages and allowed to mate. Grape juice agar plates and small amount of live yeast paste were supplied. Embryos were collected in <24 h and washed in PBS. Embryos then were seeded ∼20 μL per bottle to achieve standardised larval density. After emerging as adults, experimental flies were allowed to mate for 48 h and were split into 15 flies/vial. Vials were kept in DrosoFlippers (drosoflipper.com) and flies were transferred to fresh food 3 times a week. For RU + condition, vials contained SYA with 200 μM RU^486^ (Sigma, #M8046). Vials of control condition (RU-) contained SYA with the equivalent volume of EtOH vehicle. For the lifespan assays, deaths and sensors were scored each time the flies were transferred to the fresh food until all flies were dead or censored. Log rank tests were performed in Microsoft Excel to determine the difference between survival curves, median survival; Cox Proportional Hazards analyses were performed in R package Survival to determine the effect size of interventions and interaction between *dfoxo* and *Xbp1*^*RNAi*^. For other experiments, flies were maintained in the same setup until day 9 (after 7 days of RU+/RU- treatment), when flies were dissected to harvest the specific tissues for qPCR, western blots, or RNA-sequencing, unless indicated otherwise. For starvation assays, flies were transferred to vials containing 1% agar only. The vials were check for dead flies once or twice a day until all flies were dead.

#### Negative geotaxis assays

Flies were transferred to empty vials in DrosoFlipper at the indicated ages. The setup allowed flies to climb freely within the space of two-vial height. The same cohort was continuously assayed throughout the experiment. Flies were placed in the recording room, acclimatising for 30 min. Flies were tipped to the bottom of the vial and allowed to climb freely. The climbing behavior was video recorded for 20s. Videos were analyzed in Freeclimber (Spierer et al., 2021) to measure the positions of flies. The coordinates of flies at the same time point for each age (15 s; the time point when most young flies reach the top of the vials) were extracted and exported to R (R Core Team). Data were analyzed with a linear model (lm) and Student’s t test.

#### Developmental lethality assays

Crosses were set up in vials with SYA and allowed to mate for 2 days. Then flies were tipped to new vials and allow to lay eggs for another 24 h. The number of eggs within the new vials were counted and all adult flies were removed. After 10 days, the number of emerged adult female and male flies were counted. The vials were monitored until day 13 in case of developmental delay. The developmental lethality was counted by diving the number of emerged adults by the number of counted eggs. Flies with balancer would not carry the chromosome with the transgene of interest so were excluded from the counting, and the corresponding proportion of eggs were removed.

#### Western blotting

Proteins were extracted from dissected fat bodies (5 per samples) or S2 cells (5 mL) in 200 μL ice-cold 12.5% trichloroacetic acid. Fat body samples were first homogenised with pestle. Samples were spun at 14,000 rpm, 4°C for 15mins. Pellets were washed with 1M Tris twice before resuspending in 50 μL of sample buffer (50% LDS sample buffer, 100 mM DTT, in nuclease-free water). The samples were separated in the NuPAGE 10%, Bis-Tris gel (ThermoFisher #NP0316BOX), and transferred to a nitrocellulose blotting membrane (GE Healthcare Life Sciences #10600003) using Trans-Blot Turbo Transfer System. Membranes were blocked with 5% skimmed milk (Millipore #70166) for 1 h at room temperate and incubated with primary antibodies (anti-dFOXO 1:5000, anti-FLAG 1:2000, anti-Actin 1:1000) in 5% skimmed milk at 4°C overnight. After rinsing and washing with 0.1% Triton X-100 in PBS (PBST), membranes were probed with secondary antibodies (anti-mouse secondary antibody 1:10000 or anti-rabbit secondary antibody 1:10000) for 1 h at room temperature. After washing, membranes were stained with Crescendo (Merck #WBLUR0500), and images were captured by ImageQuantTM800 (Amersham). Densitometric analysis was done in ImageJ.

#### RNA extraction and RNA-Sequencing

Guts and fat bodies (the latter associated with the abdominal cuticle) were dissected in ice-cold PBS and placed in ice-cold Trizol (ThermoFisher Scientific #15596026). For qPCR experiments, each condition had 6 to 8 biological replicates, 5 guts or 5 fat bodies were pooled as one biological replicates. For RNA-seq experiments, each condition had 4 to 5 biological replicates, 12 guts or 12 fat bodies were pooled as one biological replicates. Sample were homogenised by ribolyser with 425–600 μm acid washed glass beads (Merck) at maximum speed for 30 s. RNA extraction was performed with Trizol and RNA concentration quantified on a NanoDrop 2000c spectrophotometer. For RNA-seq experiments, Poly(A) RNA selection, library construction, quality control and sequencing was performed by GENEWIZ (https://www.genewiz.com/en-GB/). Due to poor RNA or library yield/quality, one sample was not sequenced. 150 bp paired-end sequencing was performed using an Illumina NovaSeq instrument. 2.83 million reads per sample were achieved on average.

#### RNA-seq data processing and analysis

Read quality was assessed using FastQC. Next, reads were aligned to the *Drosophila melanogaste*r genome (dm6) using HiSAT2. Alignments were counted with featureCounts over exons per gene based on genome annotation file (r6.47). The 2 bp mutation in the reads of S106>Xbp1^U^ + RU486 sample was confirmed by Salmon. Sample clustering was performed with R function pheatmap. Samples originating from the same flies but clustering away from all others, including two S106>Xbp1^s^ gut samples, one S106>Xbp1^s^ fat body sample and one S106>wDah gut sample were removed from subsequential analysis. Differential gene expression was assessed with R package DESeq2 (Love et al., 2014). The effect of RU^486^ feeding was assessed within each genotype and tissue. Gene Ontology (GO) term analysis was performed by TopGO package in R. All the DESeq results and GO analysis results are presented in the Data S1.

#### qPCR

Following the RNA extraction described above, RNA samples were converted to cDNAs using Oligo-dT and SuperScript II Reverse Transcriptase (ThermoFisher Scientific, #18064014). Primers were supplied by Thermo Fisher. All primers applied in the study are described in the key resources table. Standard was prepared for each experiment with serial dilution of a pool of all samples. qPCR was performed on an Applied Biosystems QuantStudio 6 Flex real-time PCR instrument with Fast SYBR Green PCR Master Mix (Applied Biosciences #4385612). Relative concentration of each sample was quantified by relating to the standard curve. Tubuilin was used for normalisation in expression analysis unless its quantity was suspected of changing with one of the experimental conditions, in which case Actin was used instead. Student’s t-test was used in qPCR expression analysis.

#### Motif analysis

TF binding motifs enriched in the list (FDR 10%) of genes differentially expressed in the S106>Xbp1^s^ fat body were determined using the R package RcisTarget with all the default setting. For motif-ranking dataset, “dm6-5kb-upstream-full-tx-11species” was used. Motif-annotation dataset was the motifAnnotations_dmel embedded in the package.

For the motif co-occurrence test, genome coordinates containing either the Xbp1^s^-bound motif (ACGT) or the dFOXO-bound motif (TGTTT) were obtained from *Drosophila melanogaster* genome (dm6) with R package Biostrings. Coordinates were extended 100 bp on each side to create the 204 bp or 205 bp peak collections for co-occurrence calculation. Number of overlapping peaks between the two profiles were counted. To determine the significance of the overlap, bootstrapping analysis of the co-occurrence were conducted in R package RegioneR (Gel et al., 2015), 1000 permutation tests were conducted to calculate the likelihood of co-occurrence and the *p* value.

#### Molecular cloning

*FLAG-Xbp1u* and *FLAG-Xbp1U* were cloned into pAFW vector (pAFW-Actin5Cpromoter- 3xFLAG, DGRC_1111) as follows. Sequence of *Xbp1u* and *Xbp1U* were amplified from *UAS-Xbp1RA* and *UAS-Xbp1U* genomic DNA with the Phusion High–Fidelity DNA Polymerase (ThermoFisher Scientific #F530L), cloned into the pENTR/D-TOPO gateway plasmid (ThermoFisher Scientific #450218) following manufacturer instructions and confirmed by sequencing. Then both plasmids were recombined into the pAFW construct with Gateway LR Clonase II Enzyme Mix (ThermoFisher Scientific #11791-020) following manufacturer instructions. The constructed *Act5Cpromoter-Flag-Xbp1U* and *Act5Cpromoter* -Flag-Xbp1u plasmids were confirmed by sequencing before transfecting the S2 cells.

#### S2 cell culture

Drosophila S2 cells were cultured in Schneider’s medium (Gibco/Thermo Scientific #21720024) supplied with 10% Fetal Bovine Serum (Gibco/Thermo Scientific #A3160801). Cells were maintained in 25°C incubator with 0% CO_2_ concentration, and split with fresh media every week. Before transfection, cells were split into fresh media to reach density of ∼10^6^/mL and grown for 24 h. Transfection were conducted with the Effectne Transfection reagent (Qiagen #301425) within 12-well plates following manufacturer instructions. After transfection, cells were maintained in the media for 6 days before harvesting for protein collection.

### Quantification and statistical analysis

All statistical analyses were performed in Excel (Microsoft) or R. The statistical tests applied to each experiment are mentioned in the corresponding Figure caption or Results section. Additional information is presented in Methods. For all figures, the n number can be found in the figure legends, which corresponds to the number of biological repeats included in the experiment. For RNA-seq analyses and Motif analyses, false discovery rate [FDR] were applied to adjust for multiple testing. FDR <0.1 were considered significant. For other experiments, *p* < 0.05 were considered significant. All regression models had a fully factorial design. Asterisks indicate: ∗*p* < 0.05, ∗∗*p* < 0.01, ∗∗∗*p* < 0.001.

## Supplemental information

Document S1. Figures S1–S6

Data S1. Source data and analyzed data for all the main Figures and the related Supplementary Figures
