# Supplementary material for: Xbp1 targets canonical UPRER and non-canonical pathways in separate tissues to promote longevity
Source: iScience. 2024 May 11;27(6):109962. doi: 10.1016/j.isci.2024.109962 (PMC11144730; doi:10.1016/j.isci.2024.109962)
Supplement: Document S1. Figures S1–S6 [file mmc1.pdf]

iScience, Volume *27*

Supplemental information

Xbp1 targets canonical UPR^{ER} and non-canonical pathways in separate tissues to promote longevity

Mengjia Li, Haocheng Shou, Guillermo Martínez Corrales, Tatiana Svermova, Alessandra Vieira Franco, and Nazif Alic

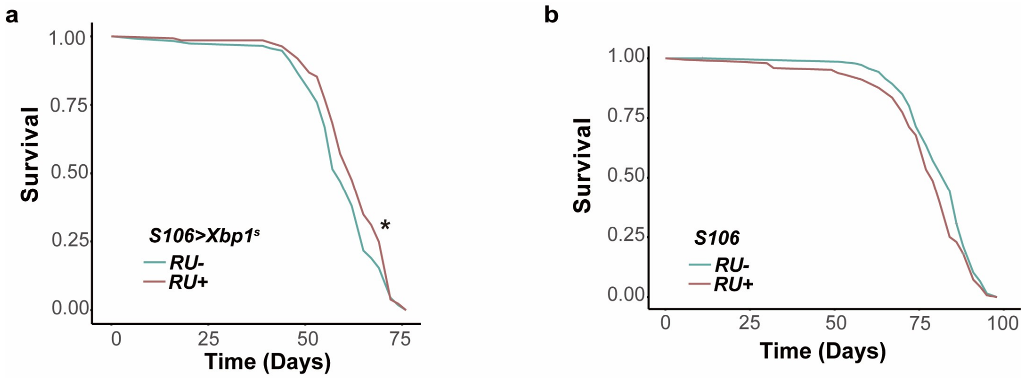

Supplementary Figure 1. Control lifespan for gut and fat body *Xbp1ˢ* overexpression, Related to Figure 1

a Another lifespan assay trail of *S106>Xbp1ˢ* females. RU^{486}- (control) n = 111 dead/ 9 censored flies, RU^{486}+ n = 132 dead/ 5 censored flies, median lifespan extension by *Xbp1ˢ* induction +5.1%, p =0.037, *log-rank test*. **b** Lifespan assays of control females carrying the *S106* driver alone, related to Figure 1a. RU^{486}- (control) n = 139 dead/3 censored flies, RU^{486}+ n = 144 dead/2 censored flies, p = 0.057, *log-rank test*. * *p<0.05*

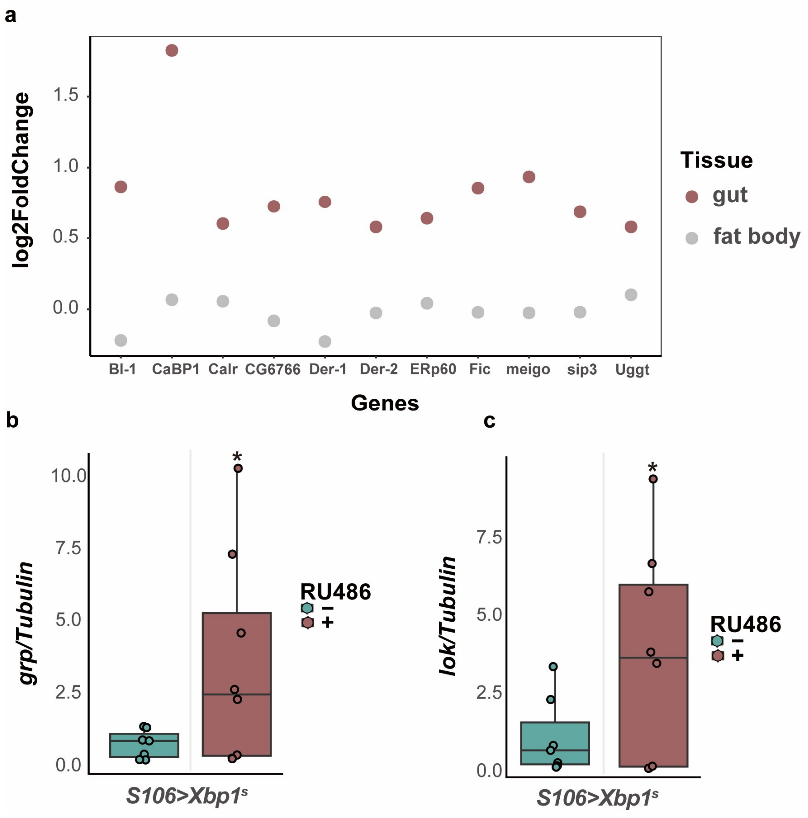

Supplementary Figure 2. Additional qPCR and gene expression comparison between gut and fat body, Related to Figure 2

a Log_2FC for genes involved in response to endoplasmic reticulum stress (GO:0034976) and whose expression was significantly altered by *Xbp1ˢ* induction in the gut. None of them were significantly altered by *Xbp1ˢ* induction in the fat body. **b** qPCR quantification of *grp* transcript upon *Xbp1ˢ* induction in fat body (n = 7). p = 0.037, *one-tailed student t-test.* **c** qPCR quantification of *lok* transcript upon *Xbp1ˢ* induction in fat body (n = 7). p = 0.040, *one-tailed student t-test. * p<0.05*

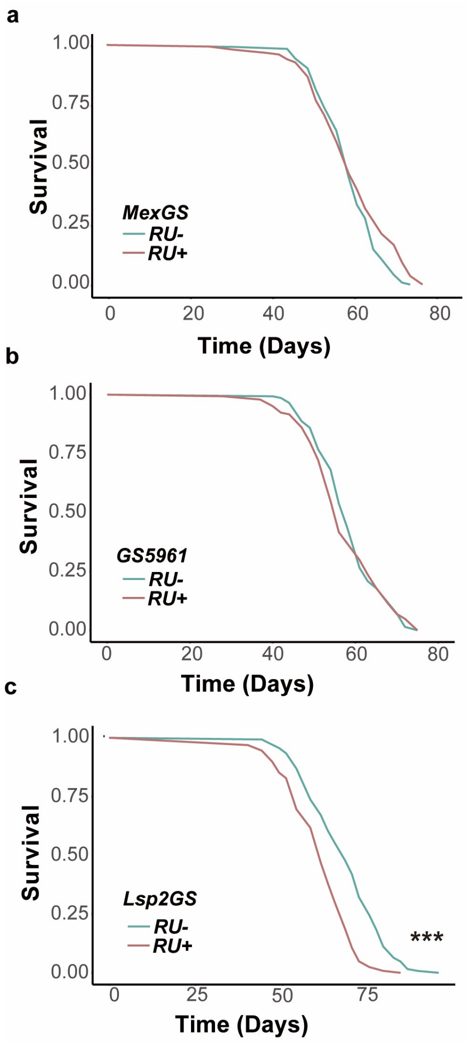

Supplementary Figure 3. Control lifespans for tissue-specific *Xbp1^s* overexpression, Related to Figure 3

a Lifespan assays of *MexGS* driver-alone control females fed or not RU^{486}. RU^{486}- (control) n = 123 dead/0 censored flies, RU^{486}+ n = 150 dead/1 censored flies, p = 0.040, *log- rank test*. **b** Lifespan assays of *GS5961* driver-alone control females. RU^{486}- (control) n = 140 dead/4 censored flies, RU^{486}+ n = 144 dead/0 censored flies, p = 0.56, *log-rank test*. **c** Lifespan assays of *Lsp2GS* driver-alone control females. RU^{486}- (control) n = 133 dead/4 censored flies, RU^{486}+ n = 126 dead/2 censored flies, p = 1.17 x 10^{-19}, *log-rank test*. *** *p<0.001*

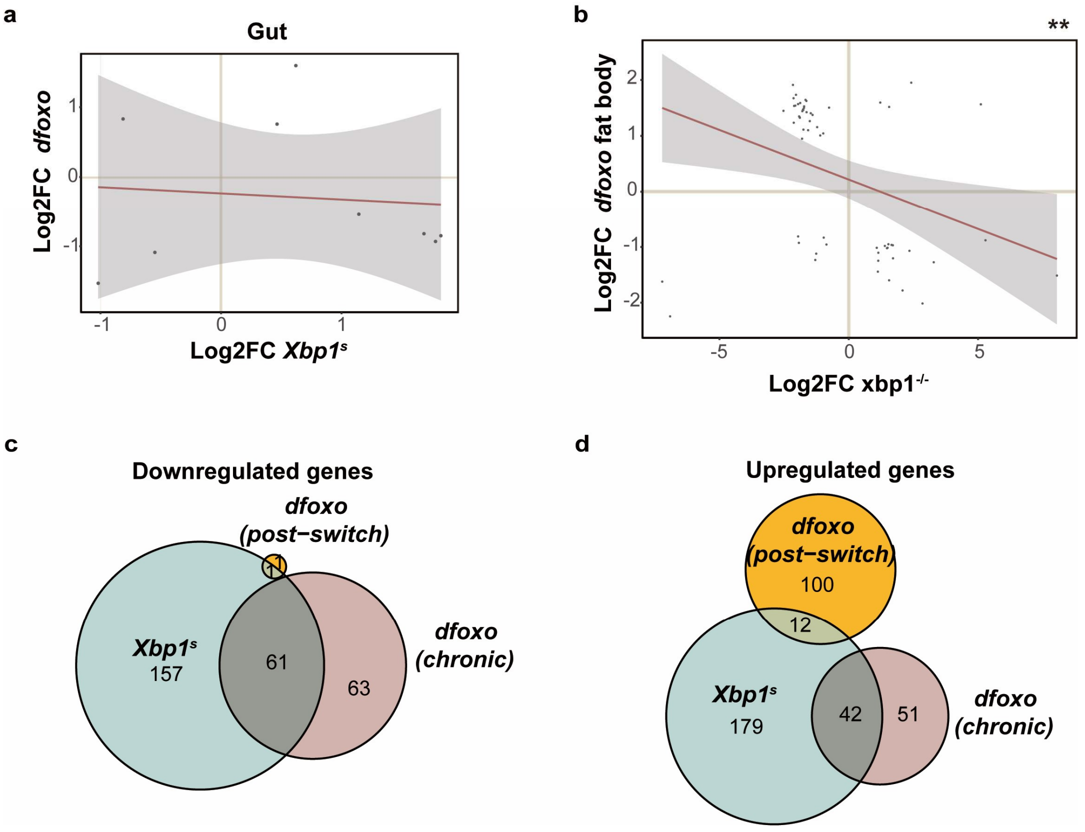

Supplementary Figure 4. Additional qPCR and western blot membranes related to Xbp1^s-dFOXO interplay, Related to Figure 4

a Relationship between log_2Fold Change (FC) in gene expression caused by *Xbp1^s* induction and that caused by *dfoxo* induction in the gut. There is no obvious correlation for genes differentially expressed in both conditions (*linear regression*, β = -0.099, p = 0.81). **b** Relationship between log_2Fold Change (FC) in gene expression caused by *Xbp1* null mutation (*Xbp1$^{-/-}$*) in larvae and that caused by *dfoxo* induction in the fat body (*linear regression*, β = *-0.18,* p = 0.0098). **c** Overlap genes differentially expressed and downregulated upon *Xbp1^s* induction, *dfoxo* induction (chronic), *dfoxo* post-switch in the fat body. **d** Overlap of genes differentially expressed and upregulated upon *Xbp1^s* induction, *dfoxo* induction (chronic), *dfoxo* post-switch in the fat body. ** *p<0.01*

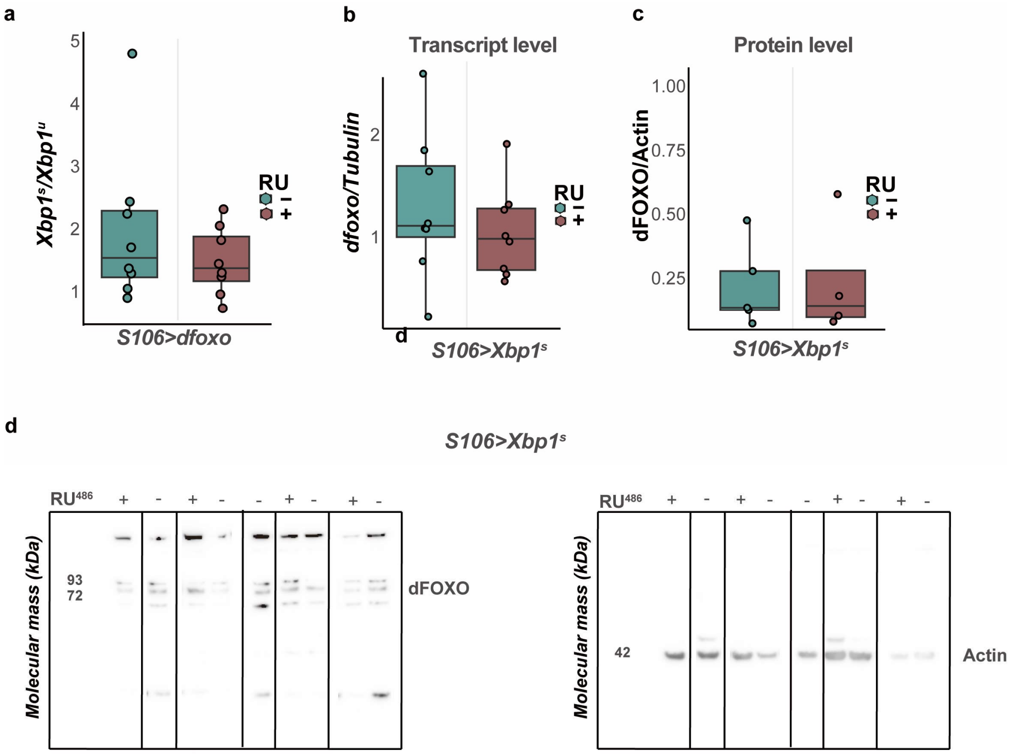

Supplementary Figure 5. Additional qPCR and western blot membranes related to Xbp1^{s}-dFOXO interplay, Related to Figure 5

a qPCR quantification of *Xbp1^{s}/Xbp1^{u}* ratio upon *dfoxo* induction in the fat body (n = 8). p =0.19, *one-tailed student t-test.* **b** qPCR quantification of *dfoxo* transcript upon *Xbp1^{s}* induction in the fat body (n = 8). p = 0.91, *one-tailed student t-test.* **c** Western-blot quantification of dFOXO upon *Xbp1^{s}* induction in the fat body ($5 \geq n \geq 4$). p = 0.30, *one-tailed student t-test.* Images of membranes are shown in Supplement Figure 5 d. **d** Images of the western-blot membranes shown in Supplement Figure 5 c. Irrelevant lanes were masked from the images as indicated.

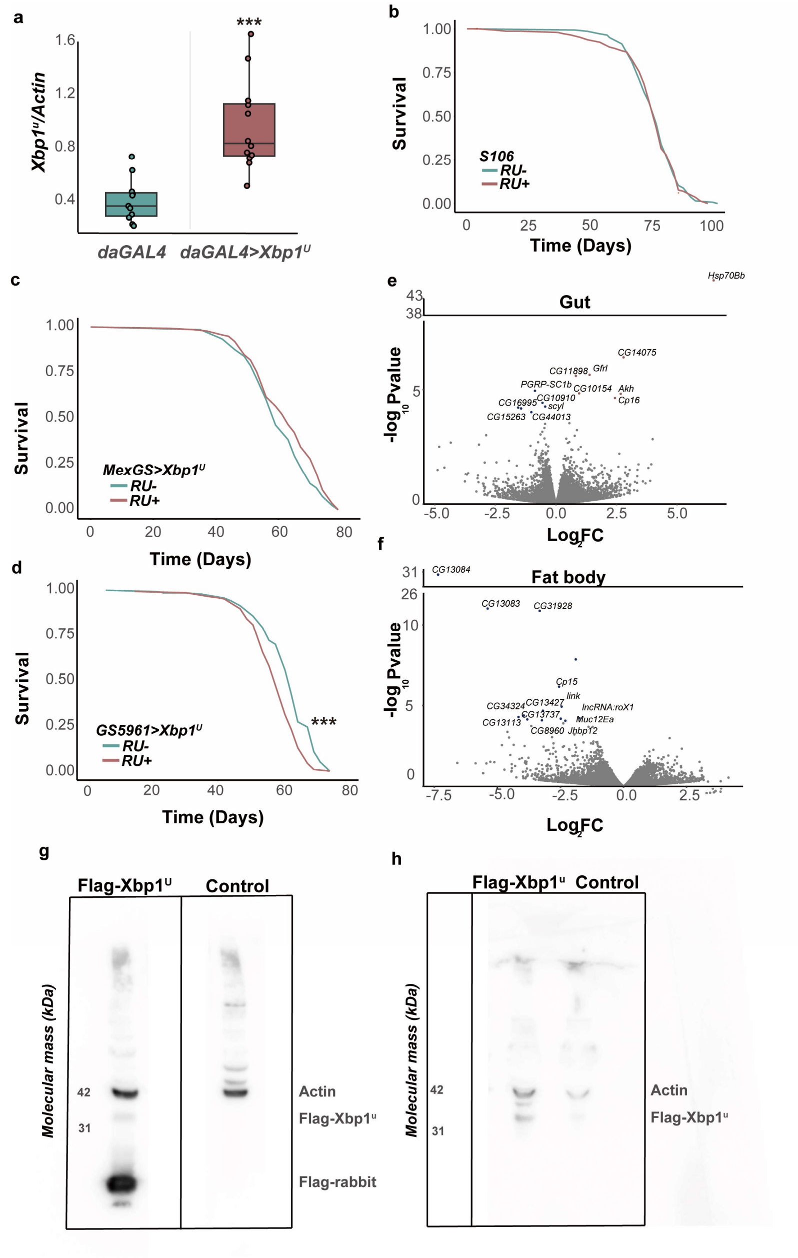

Supplementary Figure 6. Additional control lifespan, volcano plots for *Xbp1^U* RNA-seq and western blot membranes related to Xbp1^U, Related to Figure 6

a qPCR quantification of *Xbp1^u* transcripts in *Xbp1^U*-induced flies at day 9 ($12 \geq n \geq 11$), p = 5.23 x 10^{-5}, *one-tailed student t-test.* **b** Lifespan assays of *S106* driver-alone control females fed or not RU^{486}. RU^{486}- (control) n = 137 dead/2 censored flies, RU^{486}+ n = 143 dead/3 censored flies, p = 0.97, *log-rank test*. **c** Lifespan assays of *MexGS>Xbp1^U* females. RU^{486}- (control) n = 129 dead/9 censored flies, RU^{486}+ n = 139 dead/9 censored flies, p = 0.055, *log-rank test*. **d** Lifespan assays of *GS5961>Xbp1^U* females. RU^{486}- (control) n = 141 dead/1 censored flies, RU^{486}+ n = 144 dead/3 censored flies, p = 4.97 x 10^{-6}, *log-rank test*. **e** Volcano plots showing the effect of *Xbp1^U* induction on transcripts in the gut with significantly upregulated genes (FDR 10%) shown in red and significantly downregulated genes shown in blue. **f** Volcano plots showing the effect of *Xbp1^s* induction on transcripts in the fat body. **g,h** Images of the western-blot membranes shown in Figure 6 c. Irrelevant lanes were masked from the images as indicated *** *p<0.001*
